# Foveal avascular zone vessel density is associated with visual field progression in early-stage glaucoma eyes with central visual field damage

**DOI:** 10.1038/s41598-023-45541-1

**Published:** 2023-10-25

**Authors:** Jooyoung Yoon, Ko Eun Kim, Anna Lee, Woo Keun Song, Michael S. Kook

**Affiliations:** Department of Ophthalmology, Asan Medical Center, University of Ulsan College of Medicine, 88 Olympic-ro 43-gil, Songpa-gu, Seoul, 05505 Korea

**Keywords:** Glaucoma, Risk factors, Prognosis, Prognostic markers

## Abstract

We investigated the relationship between foveal avascular zone (FAZ)-related parameters, assessed by optical coherence tomography angiography (OCT-A), and visual field (VF) progression in early-stage open-angle glaucoma (OAG) eyes with central visual field (CVF) defects. Early-stage glaucoma eyes [VF mean deviation (MD) ≥ − 6 dB] with CVF defects were included. The rates of longitudinal change in FAZ-related parameters and structural parameters were evaluated and compared between VF progressors and non-progressors, using linear mixed effects models. Cox proportional hazards model and linear regression models were used to identify factors associated with VF progression, the VF MD reduction rate and the change rate of mean total deviation in central 12 VF points (MTD_10_). A total of 131 eyes were included and VF progression was detected in 32 eyes (24.4%) during 3.45 years of follow-up. The rates of reduction in vessel density in the 300 µm width annular region surrounding the FAZ (FD300) and macular ganglion cell–inner plexiform layer thickness (mGCIPLT) were significantly faster in progressors than in non-progressors. The faster VF MD or MTD_10_ reduction rates were associated with faster rates of FD300 loss and mGCIPLT reduction. The FD300 reduction rate is significantly associated with VF progression in early-stage OAG eyes with CVF defects. FD300 may be an adjunctive biomarker of VF progression in glaucomatous eyes with CVF defects.

## Introduction

Central visual field (CVF) damage has been established as having significant impact on vision-related quality of life, including reading, doing household chores, and driving^1^. It may be present in early glaucoma patients^2,3^, especially those with risk factors of vascular insufficiency, such as systemic hypotension, migraine, cold extremities, optic disc hemorrhage, and choroidal microvascular dropout^3,4^. Moreover, eyes with initial CVF defects may undergo faster global visual field (VF) progression^5^. An understanding of the pathophysiology of VF progression in patients with CVF defects is thus likely to be crucial to the effective management of glaucoma. In this regard, knowledge on the predictive factors associated with VF progression in eyes with CVF defects may provide an opportunity for more aggressive treatment of this disease, as well as enable closer monitoring during follow-up.

The foveal avascular zone (FAZ), representing the vascular status of the foveal area, is reported to be associated with microcirculatory deficiency involving the macula^6–12^, and to have correlation with visual acuity^9^ and the severity of VF damage^7,10,11^ in glaucoma patients. However, reports on the longitudinal changes of FAZ-related parameters in glaucoma patients, including the FAZ area and perimeter and foveal density 300 µm (FD300), are currently limited and no studies have yet characterized the relationship between alterations of FAZ-related parameters and subsequent glaucoma progression. Our current study evaluated progressive changes in the FAZ area, and in the perimeter and microcirculation surrounding the FAZ (i.e., FD300), in glaucoma patients with CVF defects, and their association with global VF progression. We hypothesize that progressive loss of FD300 is predictive of subsequent VF progression and is associated with faster VF progression in glaucoma patients with CVF defects.

## Materials and methods

### Subjects

The study was approved by the institutional review board of Asan Medical Center (IRB no. 2018-1008) and all procedures conformed to the tenets of the Declaration of Helsinki. The requirement for informed consent was waived by the institutional review board of Asan Medical Center due to the retrospective nature of the study design.

This longitudinal retrospective study was conducted by reviewing the medical records of the open-angle glaucoma (OAG) patients who visited the glaucoma clinic of Asan Medical Center between November 2016 and June 2022. All of these study subjects had undergone comprehensive ophthalmologic examinations including measurement of intraocular pressure (IOP) by Goldmann applanation tonometry, best-corrected visual acuity (BCVA), slit-lamp biomicroscopy, gonioscopy, axial length (AL) measurements using an IOL master (Carl Zeiss Meditec, Dublin, CA), and central corneal thickness (CCT) measured using ultrasonic pachymetry (Tomey SP-3000, Nagoya, Japan). At each follow-up, patients underwent optical coherence tomography angiography (OCT-A, AngioVue; Optovue Inc., Fremont, CA), optic nerve head (ONH)/macular imaging with spectral-domain optical coherence tomography (SD-OCT, Cirrus HD; Carl Zeiss Meditec, Dublin, CA), stereoscopic optic disc photography and red-free retinal nerve fiber layer (RNFL) photography (Canon, Tokyo, Japan), and the standard automated perimetry using Humphrey field analyzer (HFA) Swedish Interactive Threshold Algorithm (SITA)-Standard 24-2 VF testing (Carl Zeiss Meditec). The SITA-Standard 24-2 VF test was considered reliable if the following criteria were met; false-positive error < 15%, false-negative error < 15%, and fixation loss < 20%.

For inclusion in this present study, the subjects were required to satisfy each of the following criteria: (1) an age ≥ 18 years at initial presentation; (2) a BCVA of 20/30 or better; (3) a refractive error of between—6 to + 3 diopter (D) sphere, and ± 3 D cylinder; and (4) early-stage OAG. The subjects had been followed up for a minimum of two years and a maximum of five years with five or more reliable SITA-Standard 24-2 VF examinations, and good-quality SD-OCT and OCT-A measurements obtained at the same visit during follow-up, as the change in VF mean deviation (MD) becomes increasingly non-linear over time.

OAG was defined as the presence of glaucomatous ONH damage compatible with a glaucomatous VF defect, and the presence of an open angle on gonioscopy, regardless of the IOP level. A glaucomatous VF defect was confirmed based on the Anderson’s criteria^13^, detected in two consecutive reliable VF exams. A glaucomatous VF defect as defined as follows: (1) ≥ 3 adjacent points with *P* < 0.05 on a pattern deviation (PD) probability map and one abnormal point with *P* < 0.01, (2) glaucoma hemifield test outside normal limits, and (3) a pattern standard deviation of *P* < 0.05 confirmed on two consecutive VF tests^13^. The presence of glaucomatous VF defects was confirmed if the abnormalities are present within the same location on two consecutive VF tests^14^. Early-stage glaucoma was defined as a VF MD of ≥ -6 dB^13^. The exclusion criteria were as follows, and these criteria were applied to all follow-up visits: (1) a history of intraocular surgery including cataract and glaucoma surgery; (2) a history of trauma; or (3) any coexisting retinal or macular vascular/degenerative/severe myopic diseases (i.e., diabetic maculopathy, retinal vein occlusion, epi-retinal membrane, myopic staphyloma, etc.) that could impair adequate OCT/OCT-A/VF testing during follow-up. The affected eye was selected in patients with unilateral disease, while one eye was randomly selected in the cases with bilateral disease, since our study had a relatively small sample size.

### Definitions of CVF damage and VF progression

Early OAG eyes that met the initial inclusion criteria were further selected depending on the presence of CVF defects at baseline. The CVF defects were confirmed based on the presence of VF defects within 10 degrees of fixation, with at least 1 VF defect point (at *P* < 0.005) within the central 10° on two consecutive baseline examinations^5^, regardless of CVF damage extension into the 10° to 24° VF area. Event- and trend-based analyses were both applied to determine overall VF progression (i.e., VF progression occurring within and/or outside the central 10° VF area) during follow-up, since OAG eyes with CVF defects may present with CVF damage extending to the 10° to 24° VF area at baseline and this study was purposed to determine the putative factors associated with fast global VF progression. Our event-based analysis defined VF progression as a progressive VF change (“likely progression”) at three or more points at the same locations in three consecutive tests^15^ determined using HFA-guided progression analysis software (GPA; Carl Zeiss Meditec). Trend-based analysis considered a significantly (*P* < 0.05) negative slope of VF MD change (expressed in dB/yr) at two consecutive tests as indicative of VF progression^16,17^. The mean value of total deviation values of the 12 central 10° points (MTD_10_) were evaluated to assess the severity of CVF damage^18^.

### ONH/Macular SD-OCT and OCT-A assessments

The optic disc cube and macular scans were acquired using SD-OCT (Cirrus HD; Carl Zeiss Meditec). The optic disc cube scan evaluated a circular area of 3.46 mm in diameter, centered on the ONH, and the average circumpapillary retinal nerve fiber layer thickness (cpRNFLT) was thereby measured. The macular scan measured the average macular ganglion cell layer-inner plexiform layer thickness (mGCIPLT) from the circular region centered on the fovea, having inner horizontal and vertical diameters of 1.2 and 1 mm, respectively, and outer horizontal and vertical diameters of 4.8 and 4 mm, respectively. Only SD-OCT scans without motion artifacts or segmentation errors, and with good central fixation and signal strength > 6 were included in the analysis.

OCT-A (AngioVue; Optovue Inc.) macular scans of 6.0 × 6.0 mm^2^ were used to assess the FAZ parameters and microvasculature around the FAZ. The FAZ was defined as the capillary-free area that is enclosed by the innermost macular arcade. The AngioVue software automatically calculated the FAZ parameters based on the retinal slab. The FAZ area, FAZ perimeter, and FD300 parameters were thereby measured. The FAZ area was corrected by AL using the Littman formula, in order to adjust for the magnification effect^19^. The corrected FAZ area was calculated as the FAZ area × 3.46^2^ × 0.013062^2^ × (AL − 1.82)^2^^19^. The FD300 was defined as the vessel density (VD) of the 300 µm width annular region surrounding the FAZ in the superficial retinal layer, from the internal limiting membrane to 10 µm above the inner plexiform layer. All OCT-A parameters were extracted from the same version of AngioVue software (version 2018.1.0.43). Only good quality images with no motion artifacts, segmentation errors, localized weak signal intensities caused by vitreous floaters, or poor clarity (i.e., media opacity), and with a scan quality of > 6 were included in the analysis.

### Statistical analysis

For the comparison of baseline demographics and clinical parameters between the eyes with and without VF progression, normally distributed data were compared using an independent t-test. Mann–Whitney U tests were otherwise used after the normality of distribution was assessed using a Kolmogorov–Smirnov test. Categorical variables were compared using Chi-square tests. A linear mixed effects model was applied to evaluate the rates of change in the FAZ and in structural parameters over time, which was fitted using the fixed effects with age, scan quality, follow-up duration, number of tests, AL, baseline IOP, mean follow-up IOP, CCT, and baseline VF MD, accepting random intercepts and coefficients at the individual levels.

Cox proportional hazards models were performed to evaluate the clinical factors related to VF progression. All the variables with a *P* < 0.1 in univariable analysis were entered into the multivariable model. The clinical factors associated with the VF MD reduction rate and those associated with MTD_10_ change rate were assessed by linear regression analyses. Multivariable models were built using all the variables with a *P* < 0.1 in univariable analysis. Statistical analyses were performed with SPSS software, version 21.0 (IBM Corp, Armonk, NY). Statistical significance was defined as *P* < 0.05.

### Ethics approval

The study conformed to the tenets of the Declaration of Helsinki and was approved by the institutional review board of Asan Medical Center (IRB no. 2018-1008).

## Results

Initially, 157 eyes from 157 subjects were enrolled. Twenty-three eyes were excluded due to poor image quality of SD-OCT (eight eyes) and/or OCT-A (fifteen eyes), and additional three eyes were excluded due to having less than five SITA-Standard 24-2 VF, SD-OCT, or OCT-A test. A total of 131 eyes from 131 subjects were retrospectively evaluated and 32 of these cases (24.4%) showed VF progression throughout the mean follow up period of 3.45 ± 1.75 years. Out of 32 patients who showed VF progression, 29 patients met the event-based analysis criteria, while 24 patients were confirmed with trend-based analysis criteria. Baseline characteristics such as age, gender, AL, CCT, and IOP did not differ between the VF progressors and non-progressors (Table 1). The FAZ area and perimeter differed between these groups at both baseline and the final follow-up (*P* < 0.05 for all). The FD300 and mGCIPLT were similar in both groups at baseline (*P* = 0.113 and *P* = 0.137, respectively) but were significantly lower in progressors at the final follow-up (*P* = 0.009 and *P* = 0.004, respectively). The cpRNFLT did not differ between the two groups at either the baseline or final follow-up.

**Table 1:** Comparisons of clinical characteristics between open-angle glaucoma eyes with and without visual field (VF) progression.

	Entire cohort (n = 131)	VF progressor (n = 32)	VF non-progressor (n = 99)	*P* value
Age, years	57.31 ± 11.74	58.25 ± 14.04	57.01 ± 10.96	0.605
Gender, male/female	68/63	16/16	52/47	0.806
Axial length, mm	24.95 ± 1.52	24.77 ± 1.68	25.01 ± 1.46	0.503
Central corneal thickness, µm	531.13 ± 39.57	535.44 ± 44.76	529.72 ± 37.90	0.502
Follow-up duration, years	3.45 ± 1.75	3.62 ± 0.78	3.40 ± 0.73	0.141
Hypertension, n (%)	28 (21.4%)	9 (28.1%)	19 (19.2%)	0.366
Diabetes mellitus, n (%)	10 (7.6%)	4 (12.5%)	6 (6.1%)	0.329
Anti-glaucoma medication, n	1.54 ± 0.97	1.71 ± 1.04	1.46 ± 0.92	0.241
IOP, mmHg				
Baseline IOP, mmHg	15.51 ± 3.88	16.22 ± 4.53	15.28 ± 3.64	0.237
Follow-up mean IOP, mmHg	13.42 ± 1.55	13.48 ± 1.60	13.40 ± 1.55	0.806
Follow-up peak IOP, mmHg	16.19 ± 1.55	17.03 ± 4.73	15.92 ± 2.82	0.108
VF measurements				
Baseline VF MD, dB	−3.52 ± 1.73	−3.85 ± 1.78	−3.41 ± 1.71	0.226
Final VF MD, dB	−5.66 ± 2.92	−8.71 ± 3.42	−4.68 ± 1.90	** < 0.001**
Baseline VFI, %	88.23 ± 5.49	87.88 ± 5.96	88.34 ± 5.36	0.676
Final VFI, %	81.53 ± 9.94	72.69 ± 10.94	84.38 ± 7.70	** < 0.001**
FAZ area, mm^2^				
Baseline FAZ area, mm^2^	0.297 ± 0.105	0.331 ± 0.104	0.286 ± 0.104	**0.035**
Final FAZ area, mm^2^	0.311 ± 0.113	0.350 ± 0.148	0.298 ± 0.096	**0.023**
FAZ perimeter, mm				
Baseline FAZ perimeter, mm	2.092 ± 0.393	2.224 ± 0.396	2.048 ± 0.385	**0.027**
Final FAZ perimeter, mm	2.154 ± 0.418	2.302 ± 0.539	2.106 ± 0.360	**0.020**
FD300, %				
Baseline FD300, %	50.93 ± 6.34	52.48 ± 5.12	50.42 ± 6.64	0.113
Final FD300, %	49.47 ± 6.61	46.78 ± 8.55	50.31 ± 5.66	**0.009**
cpRNFLT, µm				
Baseline cpRNFLT, µm	71.62 ± 8.58	71.25 ± 9.25	71.74 ± 8.39	0.780
Final cpRNFLT, µm	70.55 ± 8.71	68.75 ± 9.17	71.13 ± 8.51	0.180
mGCIPLT, µm				
Baseline mGCIPLT, µm	67.19 ± 7.02	65.59 ± 8.62	67.72 ± 6.36	0.137
Final mGCIPLT, µm	65.12 ± 6.87	62.13 ± 8.19	66.09 ± 6.12	**0.004**

The numeric data were compared using an independent t-test and categorical data using a Mann–Whitney U test.

Bolded values denote a statistically significant difference between VF progressor.and non-progressor.

*IOP* intraocular pressure, *VF* visual field, *MD* mean deviation, *VFI* visual field index, *FAZ* foveal avascular zone, *FD300* foveal density 300 µm, *cpRNFLT* circumpapillary retinal nerve fibre layer thickness, *mGCIPLT* macular ganglion cell-inner plexiform layer thickness.

Table 2 presents the rates of change in the FAZ and structural parameters that were evaluated using linear mixed effects models. VF progressors showed a significant reduction in the FD300 (*P* = 0.001), cpRNFLT (*P* = 0.003), and mGCIPLT (*P* < 0.001), while the VF non-progressors showed a significant decrease in the mGCIPLT (*P* < 0.001), but not in the FD300 (*P* = 0.835) or cpRNFLT (*P* = 0.219). The rates of change in the FD300 (*P* = 0.022) and mGCIPT (*P* < 0.001) were significantly faster in the VF progressors.

**Table 2:** Comparisons of the rates of change in foveal avascular zone (FAZ) and structural parameters between eyes with and without visual field (VF) progression using linear mixed effects models.

	Entire cohort (n = 131)			VF progressor (n = 32)			VF non-progressor (n = 99)			
	Slope	95% CI	P value	Slope	95% CI	P value	Slope	95% CI	*P* value	*P* value*
FAZ area rate, mm^2^/year	0.003	0.001–0.005	**0.049**	0.004	−0.002 to 0.009	0.243	0.002	−0.001 to 0.005	0.119	0.444
FAZ perimeter rate, mm/year	0.011	0.002–0.020	**0.016**	0.014	−0.008 to 0.036	0.260	0.010	0.000 to 0.020	0.052	0.323
FD300 rate, %/year	−0.266	−0.497 to −0.035	**0.024**	−1.049	−1.632 to −0.465	**0.001**	−0.027	−0.278 to 0.225	0.835	**0.022**
cpRNFLT rate, µm/year	−0.268	−0.381 to −0.155	**0.014**	−0.432	−0.714 to –0.150	**0.003**	−0.175	−0.296 to −0.055	0.219	0.056
mGCIPLT rate, µm/year	−0.386	−0.477 to −0.295	** < 0.001**	−0.662	−0.846 to −0.478	** < 0.001**	−0.291	−0.391 to −0.191	** < 0.001**	** < 0.001**

Fixed effects with age, axial length, central corneal thickness, scan quality, follow-up period, number of tests, baseline intraocular pressure (IOP), mean follow-up IOP, baseline VF mean deviation (MD).

Bolded values denote a statistically significant difference between VF progressor and non-progressor.

*Comparisons between VF progressors and non-progressors using a linear mixed effects model. *FAZ* foveal avascular zone, *FD300* foveal density 300 µm, *cpRNFLT* circumpapillary retinal nerve fibre layer thickness, *mGCIPLT* macular ganglion cell-inner plexiform layer thickness.

The clinical factors associated with VF progression were next evaluated (Table 3) and a larger FAZ perimeter at baseline (hazards ratio [HR] 1.018, *P* = 0.045) was found to be significantly associated with VF progression in multivariable analysis. In the multivariable analysis investigating the clinical factors associated with a faster VF MD reduction rate (Table 4), a higher follow-up peak IOP (β = −0.034, *P* = 0.002), faster FD300 reduction rate (β = 0.108, *P* < 0.001) and faster mGCIPLT reduction rate (β = 0.180, *P* = 0.033) remained significantly associated with the VF MD reduction rate, after adjusting for potential confounding factors. The faster rate of MTD_10_ reduction was significantly associated with larger FAZ area at baseline (β = −0.023, *P* = 0.027), faster FD300 reduction rate (β = 0.101, *P* = 0.007) and faster mGCIPLT reduction rate (β = 0.192, *P* = 0.044).

**Table 3:** Univariable and multivariable Cox proportional hazards model analysis of clinical factors associated with visual field (VF) progression.

	Entire (n = 131)					
	Univariable analysis			Multivariable analysis		
	HR	95% CI	P value	HR	95% CI	*P* value
Age, years	1.005	0.974–1.036	0.776			
Gender	1.062	0.531–21.125	0.864			
Axial length, mm	0.905	0.684–1.198	0.486			
Central corneal thickness, µm	1.003	0.993–1.013	0.538			
Baseline IOP, mmHg	1.045	0.968–1.129	0.260			
Baseline VF MD, dB	0.905	0.744–1.101	0.319			
Baseline FAZ area (× 100), mm^2^	1.025	0.997–1.054	0.080	1.057	0.994–1.125	0.087
Baseline FAZ perimeter (× 100), mm	1.009	1.001–1.017	**0.030**	1.018	1.000–1.037	**0.045**
Baseline FD300, %	1.043	0.981–1.108	0.176			
Baseline cpRNFLT, µm	0.998	0.959–1.040	0.938			
Baseline mGCIPLT, µm	0.963	0.916–1.012	0.136			

The values for the foveal avascular zone (FAZ) area and perimeter were multiplied by 100 to enhance the readability of the table. Bolded values denote a statistically significant difference.

*HR* hazard ratio, *IOP* intraocular pressure, *VF* visual field, *MD* mean deviation, *FAZ* foveal avascular zone, *FD300* foveal density 300 µm, *cpRNFLT* circumpapillary retinal nerve fibre layer thickness, *mGCIPLT* macular ganglion cell-inner plexiform layer thickness.

**Table 4:** Univariable and multivariable linear regression analysis of clinical factors associated with the visual field mean deviation (VF MD) reduction rate and the change rate of mean total deviation values within the central 10° visual field (MTD_10_).

	Entire (n = 131)					
	Univariable analysis			Multivariable analysis		
	β	95% CI	*P* value	β	95% CI	*P* value
Factors associated with the change rate of visual field mean deviation						
Age, years	−0.001	−0.008 to 0.006	0.739			
Gender	0.042	0.126 to 0.210	0.625			
Axial length, mm	−0.008	−0.077 to 0.061	0.824			
Central corneal thickness, µm	−0.001	−0.003 to 0.001	0.474			
Baseline IOP, mmHg	−0.010	−0.031 to 0.012	0.374			
Follow-up mean IOP, mmHg	−0.034	−0.008 to 0.020	0.217			
Follow-up peak IOP, mmHg	−0.036	−0.060 to -0.012	**0.004**	−0.034	−0.056 to −0.012	**0.002**
Baseline VF MD, dB	−0.022	-0.070 to 0.027	0.378			
Baseline FAZ area (× 100), mm^2^	−0.006	−0.013 to 0.001	0.117			
Baseline FAZ perimeter (× 100), mm	−0.002	−0.004 to 0.000	0.092	−0.001	−0.003 to 0.001	0.351
Baseline FD300, %	−0.003	−0.017 to 0.010	0.642			
Baseline cpRNFLT, µm	0.002	−0.008 to 0.012	0.639			
Baseline mGCIPLT, µm	0.006	−0.007 to 0.018	0.362			
FAZ area rate (× 100), mm^2^/year	−0.011	−0.071 to 0.050	0.722			
FAZ perimeter rate (× 100), mm/year	−0.003	−0.018 to 0.013	0.736			
FD300 rate, %/year	0.092	0.031 to 0.153	**0.002**	0.108	0.056 to 0.161	** < 0.001**
cpRNFLT rate, µm/year	0.150	0.013 to 0.286	**0.004**	0.163	0.024 to 0.301	0.054
mGCIPLT rate, µm/year	0.306	0.159 to 0.452	** < 0.001**	0.180	0.390 to 0.320	**0.033**
Factors associated with the change rate of mean total deviation values within the central 10° visual field (MTD_10_)						
Age, years	0.002	−0.006 to 0.011	0.603			
Gender	0.143	−0.057 to 0.343	0.159			
Axial length, mm	−0.054	−0.130 to 0.021	0.156			
Central corneal thickness, µm	−0.003	−0.005 to 0.000	**0.034**	−0.002	−0.005 to 0.000	0.084
Baseline IOP, mmHg	−0.013	−0.039 to 0.013	0.327			
Follow-up mean IOP, mmHg	−0.023	−0.088 to 0.042	0.484			
Follow-up peak IOP, mmHg	−0.030	−0.060 to -0.001	**0.042**	−0.031	−0.055 to 0.000	0.066
Baseline VF MD, dB	−0.029	−0.087 to 0.029	0.329			
Baseline FAZ area (× 100), mm^2^	−0.011	−0.020 to -0.002	**0.014**	−0.023	−0.043 to −0.002	**0.027**
Baseline FAZ perimeter (× 100), mm	−0.002	−0.005 to 0.000	0.070	0.005	−0.001 to 0.010	0.108
Baseline FD300, %	−0.008	−0.024 to 0.008	0.342			
Baseline cpRNFLT, µm	0.007	−0.005 to 0.019	0.225			
Baseline mGCIPLT, µm	0.013	−0.001 to 0.027	0.075	0.013	−0.002 to 0.027	0.081
FAZ area rate (× 100), mm^2^/yr	−0.020	−0.092 to 0.052	0.587			
FAZ perimeter rate (× 100), mm/yr	−0.006	-0.025 to 0.012	0.491			
FD300 rate, %/yr	0.108	0.035 to 0.181	**0.004**	0.101	0.028 to 0.174	**0.007**
cpRNFLT rate, µm/yr	0.201	0.031 to 0.370	**0.014**	0.119	−0.071 to 0.309	0.219
mGCIPLT rate, µm/yr	0.234	0.052 to 0.417	**0.012**	0.192	0.006 to 0.378	**0.044**

The values for the foveal avascular zone (FAZ) area and perimeter were multiplied by 100 to enhance the readability of the table.

Bolded values denote a statistically significant difference.

*IOP* intraocular pressure, *VF* visual field, *MD* mean deviation, *FAZ* foveal avascular zone, *FD300* foveal density 300 µm, *cpRNFLT* circumpapillary retinal nerve fibre layer thickness, *mGCIPLT* macular ganglion cell-inner plexiform layer thickness.

**Figure 1:**
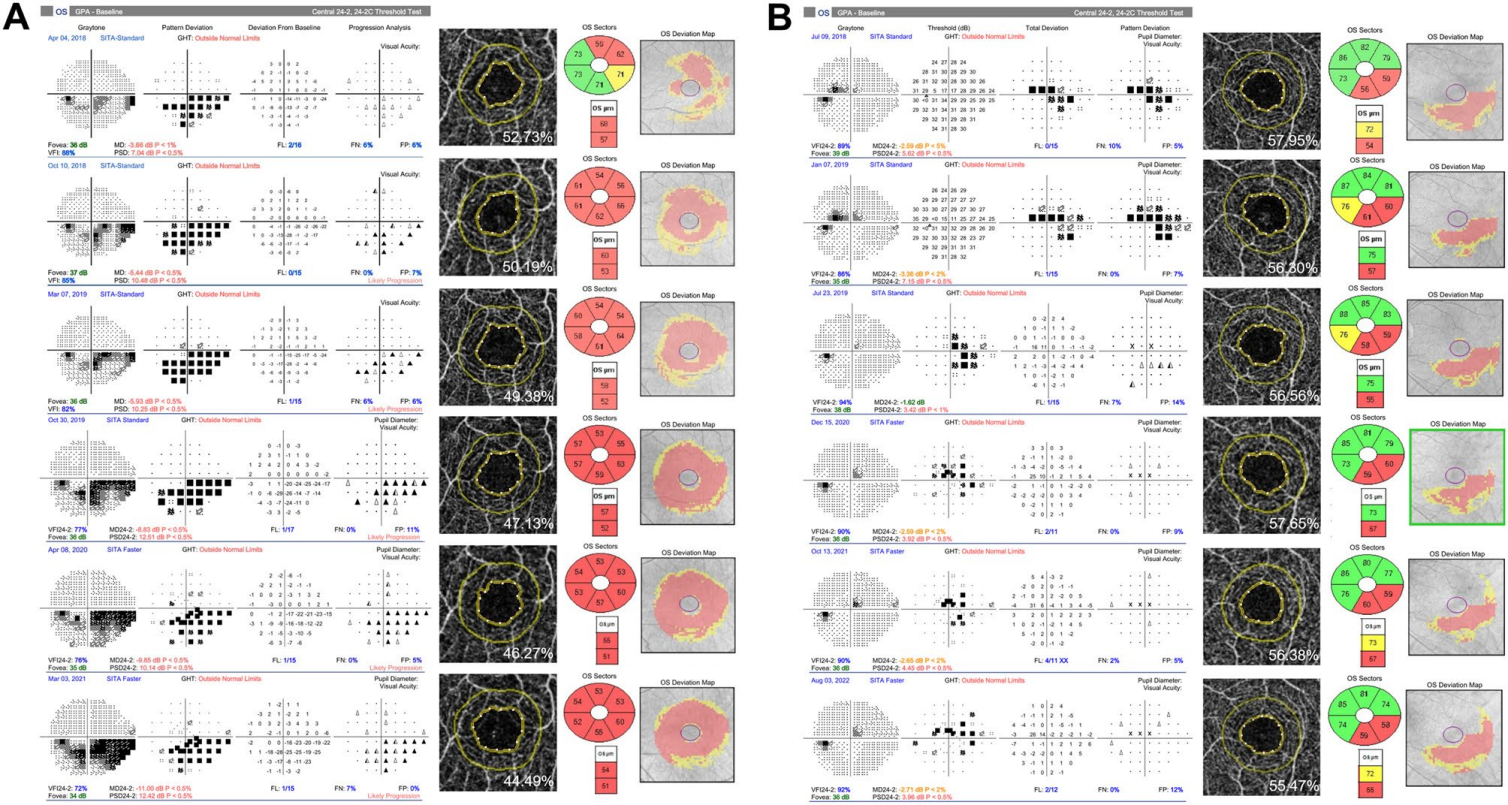
Two representative cases (**A,B**) of early-stage open-angle glaucoma eyes with central visual field defects with and without subsequent visual field progression. The progressive case (case A) showed significant longitudinal decreases in FD300 and macular ganglion cell inner plexiform layer thickness (mGCIPLT) in association with visual field deterioration, whereas the nonprogressive case (case B) with visual field testing showed no significant changes in either the FD300 or mGCIPLT during follow-up.

Figure 1 shows representative cases in our present study cohort of early-stage OAG eyes with CVF damage with and without VF progression. Figure 1A presents the observed correlations between FD300 and mGCIPLT reductions over time with concurrent VF progression, while Fig. 1B illustrates that no significant change was evident in either the FD300 or mGCIPLT over time in the case with a stable VF.

## Discussion

We have observed in our present study that the development and the rate of VF progression are significantly associated with the FD300 and mGCIPLT reduction rates in early OAG eyes with CVF defects. The baseline FAZ perimeter was related to the odds of a VF progression event, whereas the FAZ area at baseline showed an association with the speed of CVF progression. These results imply that the foveal microcirculation evaluated by measuring the FD300 and FAZ area/perimeter could be used as an adjunctive biomarker to assess glaucoma progression in eyes with CVF defects. To our knowledge, our present study is the first to assess the relationship between the rates of change in FAZ-related microcirculation and subsequent VF progression in early-stage OAG patients presenting with CVF damage at baseline.

The FAZ depicts the vascular perfusion status of the foveal region. The destruction of the foveal vascular arcade induces the enlargement of the FAZ area^6,8,12^ and increases the irregular outline of the FAZ, resulting in a larger FAZ perimeter. Thus, an increased FAZ area or perimeter has been considered as one of the good indicators of vascular dropout and capillary non-perfusion^12^. The enlargement of these parameters has been reported previously under ischemic conditions such as diabetic retinopathy^8^ and retinal vascular occlusion^6,12^, and in glaucomatous eyes with CVF defects^10,11^.

In our present study series, the high likelihood of VF progression showed an association with a large FAZ perimeter at baseline, and a faster CVF progression rate was related to a large baseline FAZ area. These findings indicated that eyes with more severe foveal vascular insufficiency and having a larger FAZ perimeter or area at baseline are prone to undergoing more rapid overall VF or CVF destruction. These findings may be in line with previously reported evidence that the area of the FAZ increases according to the severity of glaucomatous damage^7,10,11^ and that a lower macular VD is associated with longitudinal VF progression^20,21^. Hence, glaucoma patients with a relatively large FAZ area or perimeter at baseline should receive careful monitoring for subsequent VF progression, including CVF area.

It is interesting to note that the baseline FAZ area and perimeter were larger in our VF progressors, but the baseline structural and functional parameters were not significantly different between the VF progressors and non-progressors in our present cohort. This suggests that changes in FAZ parameters may be detectable prior to any alterations in structural or functional parameters in glaucoma patients with CVF defects. Vascular insufficiency is an important pathophysiology in the development of CVF defects^3^ and ischemic conditions may be present before any detectable functional loss^22^. Yarmohammadi et al.^22^ have reported that glaucomatous eyes with single-hemifield VF damage have a diffuse decrease in the peripapillary and macular VD, indicating widespread vascular damage in their perimetrically intact hemiretinae. The macular VD was reported by Penteado et al. to outperform ganglion cell complex (GCC) thickness in detecting the presence of glaucomatous damage^23^. Possible explanations for these results could be a stronger role of vascular factors in glaucomatous damage^3,4^, or the inclusion of non-neuronal component in OCT parameters which may cause artifacts or variability in assessing the true damage of retinal nerve fiber thickness^24^. In short, our current results suggest that the FAZ area and perimeter are sensitive biomarkers that reflect glaucomatous damage, particularly involving the CVF.

In our present study cohort, the FD300 decreased significantly faster in the VF progressor group and this reduction rate correlated with the rate of subsequent VF and CVF progression. FD300 is the VD measured at a distance of 300 µm from the FAZ and the strength of this parameter is that it measures the vascularity that supports only the macular ganglion cells. In the foveal area, a single layer of capillary arcade, forming the border of the FAZ^25^, continues away from the fovea until the retinal peripapillary capillaries (RPCs) appear in the perifoveal region. Since almost no RNFL exists in the foveal region, RPCs, which preferentially support the RNFL and are present in proportion to the RNFL distribution^25,26^, do not exist in the area of 0.9 ± 0.1 mm from the fovea^26^. The FD300, on the other hand, measures the VD of 300 µm width annular region surrounding the FAZ border, making its measurement sensitive to VF progression in the CVF area. Our present results showing a significant correlation between the FD300 and VF progression are consistent with previous findings which demonstrated that the FD300 represents the perfusion status of RGCs for foveal function and is significantly correlated with CVF MD^27^. Hence, the FD300 may be a useful parameter for monitoring glaucoma progression, especially in patients with CVF defects.

The reduction rate of the mGCIPLT was also significantly faster in the VF progressors in our present cohort and the increased rate of mGCIPLT loss was significantly associated with the speed of VF progression. It is well established that glaucoma induces the loss of macular RGCs^10,11,28,29^. Due to the central concentration of RGCs in the macular area, with more than 30% of these cells being located in the macula and mainly in the central 4.5 mm diameter region^30^, the loss of the mGCIPLT is more distinctive than that of the cpRNFLT in glaucoma eyes with CVF defects^28^. The degree of GCC thinning is correlated to the severity of glaucoma and more prominent in early glaucoma patients^31^. Considering that our present study subjects comprised early-stage glaucoma patients with CVF defects, it was not surprising to us to observe a close association between VF progression rate and the increased rate of mGCIPLT loss. Although the reduction rate of mGCIPLT in our VF progressors was significantly higher, our OAG patients without VF progression also showed a significant mGCIPLT loss over time. This may be explained by the discordance of structural and functional progression^32^ and/or age-related degeneration of the macular RGCs^33^.

Similar to our current investigation, Wu et al.^34^ have previously demonstrated that a loss of macular VD and GCC are associated with a faster rate of past CVF progression in a 10-2 VF test and that a lower macular VD showed a correlation with the increased odds of prior CVF progression. Nonetheless, these two studies differ in that we performed structural/foveal microcirculation/functional assessments in a longitudinal manner and evaluated the progression of glaucomatous VF loss concurrently with the change of OCT/OCT-A parameters. Our present study has demonstrated that the increased rates of FD300 and mGCIPLT loss, not the baseline parameters, were significantly correlated to the rate of VF progression. These findings are clinically relevant by suggesting that longitudinal decreases in the FD300 and mGCIPLT, rather than cross-sectional assessments, may provide more valuable information regarding glaucoma progression.

In our current study also, the cpRNFLT reduction rate was not found to be associated with VF progression, despite the fact that this progression was determined regardless of CVF damage extending into 10° to 24° VF area. This result may be due to our study cohort consisting of early OAG eyes (MD = −3.50 dB) with CVF damage. Early glaucoma patients with parafoveal VF loss are known to have more prominent damage at the macular RGC layers than the circumpapillary retinal nerve fiber layer (cpRNFL)^29^, and CVF progression is known to be more related to a loss of mGCIPLT, rather than of cpRNFLT, due to a topographic discordance between the cpRNFL and central visual function^28^ . Our recruitment of patients with early glaucomatous CVF damage may have resulted in a lack of significant association between cpRNFLT reduction and subsequent VF progression.

The peak IOP during follow-up in our present series indicated a linear association with the rate of global VF progression. The VF reduction rate was found to be independently associated with the peak IOP during follow-up in our current analysis, even after controlling for the effects of the loss of mGCIPLT and FD300. This result supports the widespread notion that the peak IOP is a risk factor for VF progression^35^ and that controlling the peak IOP during follow-up may alleviate the fast progression of glaucoma.

Our present study had several limitations of note. First, only early-stage OAG eyes with CVF defects were evaluated and other glaucoma patients with only peripheral VF defects may show different outcomes. Previous studies have shown the correlation of FAZ parameters with the presence or severity of CVF defects, rather than peripheral VF defects, due to topographic association between fovea area and CVF^10^. Therefore, our study subjects were limited to those with CVF defects in order to better elucidate the association between the changes in FAZ parameters and VF progression, but further studies are needed to explore whether these parameters are applicable to all glaucoma patients. Second, relatively small sample size may also limit the generalizability of our study findings to the glaucoma population. Third, the FAZ-related VD parameters provided by OCT-A might not completely represent the anatomical vascularity of the FAZ. OCT-A signals rely on the red blood cell (RBC) density and velocity. The RBCs move at variable velocities according to the metabolic demand^36^, which may even pause occasionally^37^. This may have resulted in an inaccurate detection of single-layer capillary microcirculation around the FAZ in our present analyses. Fourth, only FAZ-related VD parameters (i.e., the FAZ area and perimeter, and FD300) in the macula were here investigated as it has been already reported that a lower macular VD is significantly associated with longitudinal VF progression^20,21^. Fifth, VF progression was not defined by VF progression within the central 10° area but by global VF progression, despite the inclusion of OAG eyes with CVF damage in our cohort. This is because our aim was to determine the clinical factors associated with rapid global VF progression. Instead, the analyses on factors associated with the faster rate of MTD_10_ reduction was performed in order to evaluate the relationship between each parameter and CVF progression. Sixth, the association between rates of FD300 and mGCIPLT loss and glaucoma progression cannot be directly demonstrated in this study due to the lack of independent reference standard for progression. Moreover, due to the small sample size of VF progressors (n = 32), we were not able to properly analyze and provide which parameter (FD300 vs. mGCIPLT) preceded in terms of significant change between FD300 and mGCIPLT. Lastly, ocular or systemic pressure-lowering medications may have influenced our FAZ-related VD measurements. Hence, our current findings should be cautiously interpreted considering the compounding effects of ocular and systemic medications on OCT-A measurements.

In conclusion, a faster FD300 reduction indicates a higher risk of faster VF progression in early OAG patients with CVF defects. An enlarged FAZ perimeter and area at baseline are also predictive factors for VF progression and its speed, respectively. The longitudinal loss of foveal vascularity, such as FD300, may be an useful adjunctive parameter for evaluating VF progression in OAG patients with CVF defects.

## Data Availability

Data are available from the corresponding author upon reasonable request. All data relevant to the study are included in the article.
